# Essential Oil of *Croton ceanothifolius* Baill. Potentiates the Effect of Antibiotics against Multiresistant Bacteria

**DOI:** 10.3390/antibiotics9010027

**Published:** 2020-01-14

**Authors:** Ana C. J. de Araújo, Priscilla R. Freitas, Cristina Rodrigues dos Santos Barbosa, Débora F. Muniz, Janaína Esmeraldo Rocha, José B. de Araújo Neto, Maria M. C. da Silva, Talysson F. Moura, Raimundo L. S. Pereira, Jaime Ribeiro-Filho, Luiz E. da Silva, Wanderlei do Amaral, Cícero Deschamps, Saulo R. Tintino, Marcello Iriti, Sara Vitalini, Henrique D. Melo Coutinho

**Affiliations:** 1 Department of Biological Chemistry, Regional University of Cariri, Crato 63105-000, Brazil; caroljustino@outlook.com (A.C.J.d.A.); priscilla.r.freitas@hotmail.com (P.R.F.); cristinase75@gmail.com (C.R.d.S.B.); deehmuniz78@gmail.com (D.F.M.); janainaesmeraldo@gmail.com (J.E.R.); jose.bezerra456@gmail.com (J.B.d.A.N.); f.milena.silva2000@gmail.com (M.M.C.d.S.); talyssonsilva@yahoo.com.br (T.F.M.); raimundoluizbio@gmail.com (R.L.S.P.); saulorelison@gmail.com (S.R.T.); hdmcoutinho@gmail.com (H.D.M.C.); 2 Gonçalo Moniz Institute, Fundação Oswaldo Cruz (IGM-FIOCRUZ/BA), Salvador 40296-710, Brazil; jaimeribeirofilho@gmail.com; 3 Setor Litoral, Federal University of Paraná, Curitiba 80060-000, Brazil; luiz_everson@yahoo.de (L.E.d.S.); wdoamaral@hotmail.com (W.d.A.); cicero@ufpr.br (C.D.); 4 Department of Agricultural and Environmental Sciences, Milan State University, via G. Celoria 2, 20133 Milan, Italy; marcello.iriti@unimi.it

**Keywords:** antibiotics, bacterial resistance, *Croton ceanothifolius*, essential oils

## Abstract

This study is a pioneer in reporting the antibacterial properties of the species *Croton ceanothifolius* Baill. The genus *Croton* belongs to the family Euphorbiaceae composed of numerous species with documented biological activities. However, the pharmacological properties of *C. ceanothifolius* remain poorly understood. The leaves of this plant were submitted to hydrodistillation for essential oil (CcEO) extraction and the phytochemical characterization of the oil was performed by GC/MS. The minimum inhibitory concentration of the CcEO was determined for the evaluation of antibacterial activity against multiresistant strains of *Staphylococcus aureus*, *Pseudomonas aeruginosa,* and *Escherichia coli*. The antibiotic-modulating activity of the oil, in combination with antibiotics, was also evaluated. The combination of the CcEO with penicillin, norfloxacin, and gentamicin presented a synergistic effect. This effect was more significant for the association with antibiotics of the quinolone and aminoglycoside classes against *Escherichia coli*. The association of oil with gentamicin showed better results with regard to the Gram-positive strain. The association of the oil with norfloxacin against *P. aeruginosa* also showed synergism, but the association with penicillin did not change the effect of this antibiotic. Thus, it is concluded that *C. ceanothifolius* essential oil selectively potentiates the action of antibiotics against multiresistant strains.

## 1. Introduction

The use of plants’ therapeutic purposes is an ancient practice. The therapeutic properties of medicinal plants have been attributed to the presence of secondary metabolites [1], which, besides playing critical physiological roles in these organisms, interfere with pharmacological targets in humans and many other species. Therefore, medicinal plants are relevant sources of new molecules with potential for use in drug development [2].

The family Euphorbiaceae consists of a great diversity of widely distributed species. There are approximately 7600 species and 300 genera in this family, which, due to biological diversity, has aroused the interest of researchers worldwide [3,4]. The genus *Croton* stands out for presenting a large number of species in tropical and subtropical regions [5] and for its extensive use in traditional medicine in communities of Asia, Africa, and South America [6]. In Brazil, *Croton* species have been used mainly to treat inflammatory [7] and infectious [8,9] diseases. The popular use of this genus led to the development of scientific research to validate its actions. Some species of the genus *Croton* have been tested against multiresistant bacteria and both the direct antibacterial activity of their essential oil and the modifying activity of antibiotic action have been proven [10,11].

*Staphylococcus aureus* has been reported as one of the most common causative agents of nosocomial infections. Although commonly found in the skin and nasopharynx of healthy individuals, this microorganism can cause serious illness in immunocompromized individuals. In addition, the raise of cases of antibiotic resistance involving *S. aureus* highlights its importance in the context of multidrug resistance [10].

Resistance to antibacterial drugs has become an alarming public health problem. Recent studies have shown a growing number of infections caused by resistant bacteria, which cause infections in hospitals and communities [12]. A study by Da Silva et al. [13] analyzing more than 300 samples showed that half of them contained multi-resistant strains of *Pseudomonas aeruginosa* and *Escherichia coli*.

Therefore, given the importance of developing novel drugs to treat infections caused by multiresistant bacteria, and considering the therapeutic potential of the genus *Croton*, this study aimed to evaluate the antibacterial and modulatory activities of *Croton ceanothifolius* Baill. A phytochemical characterization was also carried out to identify the major secondary metabolites and direct future studies with isolated constituents of this species.

## 2. Results and Discussion

*C. ceanothifolius* essential oil yielded 0.23% of the total weight of dried leaves. After analysis by gas chromatography coupled to mass spectrometry (GC/MS) 25 constituents were identified, including bicyclogermacrene (26.3%), germacrene D (14.7%), and E-caryophyllene (11.7%) as major components (Table 1).

Compared to data in the literature, these results suggest that species of the genus *Croton* may have similar chemical compositions. Studies on *C. pallidulus*, *C. isabelli*, *C. ericoides* [14], *C. argyrophylloides*, and *C. sincorensis* [15] showed a predominance of sesquiterpenes as found in *C. ceanothifolius* essential oil. Moreover, these species presented the same major components, indicating that the relative composition of the constituents can also be conserved among the species of this genus. Importantly, Souza et al. [15] proved that climate variation affects the production of secondary metabolites since the concentration of specific metabolites varied as a function of seasonality. The genus *Croton* has been widely investigated in several areas of biology. However, the species *C. ceanothifolius* remains poorly studied. Thus, this study is a pioneer in the chemical characterization and identification of the pharmacological activity of this species.

Essential oils are volatile, aromatic, low molecular weight lipophilic substances. Studies have shown that, at low concentrations, these substances have antibacterial activity [16,17], besides presenting the potential to modulate antibiotic resistance. Therefore, the present study evaluated the antibacterial and modulating potential of *C. ceanothifolius* essential oil. Analyzing the Minimum Inhibitory Concentration (MIC) of CcEO against multidrug resistant bacteria, we found values greater than 1024 µg/mL against all strains evaluated, indicating that the oil did not present clinically relevant antibacterial activity. A similar result was obtained by Leite et al. [18], studying the antibacterial activity of *Croton limae* essential oil.

Studies show that natural products without antibacterial activity can potentiate the effect of certain antibiotics, which is known as synergism [19]. The *C. ceanothifolius* essential oil is characterized by the presence of bicyclogermacrene, germacrene D and caryophyllene sesquiterpenes as major compounds (Table 1). Earlier studies using the disk diffusion method showed that caryophyllene inhibited the growth of *S. aureus*, *P. aeruginosa*, and *E. coli* [20].

**Figure 1:**
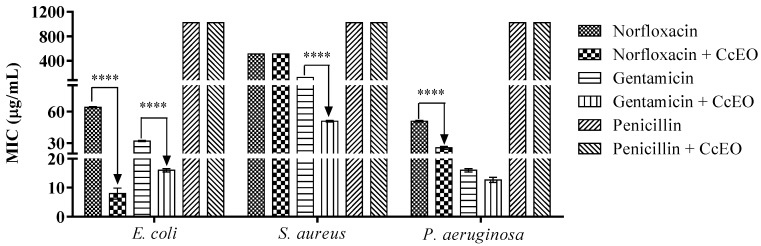
Antibiotic-Modulating effect of *Croton ceanothifolius* (CcEO) essential oil against multidrug resistant bacteria. **** Statistical Significance *p* < 0.0001.

Sesquiterpenes are known to have a direct action on the bacterial outer membrane, causing direct lysis or modifying its selective permeability. Our data, however, suggest that CcEO does not act directly, which raises the hypothesis that it may alter the membrane permeability by enhancing the effect of antibiotics [21]. As shown in Figure 1, CcEO showed an antibiotic-modulating activity that varied according to bacterial strain and type of drug.

The association of 188 µL of the essential oil with gentamicin caused a synergistic effect against all strains tested. The MIC of this antibiotic was reduced from 128 to 50 µg/mL against *S. aureus* and from 32 to 16 µg/mL against *E. coli* tests. In tests with *P. aeruginosa* CcEO decreased gentamicin MIC from 16 µg/mL to 12 µg/mL, but in this case, there was no statistical difference. Accordingly, Vidal et al. [11] demonstrated that *C. rhamnifolioides* essential oil reduced gentamicin MIC against multidrug-resistant *S. aureus* and *E. coli* strains. In addition, Da costa et al. [9] found that *C. rhamnifolioides* essential oil exhibits antibacterial activity against Gram-positive strains. *C. zehntneri* also showed modifying the effect of antibiotic action against strains of *S. aureus* and *E. coli* [22]. These studies corroborate the data obtained in the present research and highlight the genus *Croton* for its antibacterial potential.

Following the characterization of the antibiotic-modulating activity, the CcEO was tested against two Gram-negative bacteria (*E. coli* and *P. aeruginosa*) and one Gram-positive strain (*S. aureus*). Our data indicate that the oil has stronger activity against Gram-negative bacteria. Interestingly, these strains have a natural drug resistance profile due to the lipopolysaccharide layer, which hinders the permeability of these substances. Our tests demonstrated that the CcEO potentiated the action of gentamicin and norfloxacin, which act within the bacterial cell, suggesting that *C. ceanothifolius* essential oil facilitated the permeability of these drugs to the bacterial cytoplasm [23].

On the other hand, CcEO failed to modulate penicillin activity under all conditions tested, suggesting an effect that is related to the mechanism of action of this antibiotic. In this case, bacterial resistance is caused by the action of β-lactamases, enzymes that hydrolyze the functional component of penicillin-class drugs. Many bacteria today have acquired this resistance mechanism mainly due to the indiscriminate use of these antibiotics [24]. Here, we suggest that while CcEO has facilitated drug entry (due to increased permeability), penicillinases continue to degrade its β-lactam ring, causing enzymatic inactivation.

## 3. Materials and Methods

### 3.1. Plant Material

Leaves of *Croton* specimens were collected in the Butuguara Private Natural Heritage Reserve (RPPN) in the municipality of Palmeira, PR, located at 25°20.884′ S and 49°47.258′ W. This region has altitudes ranging from 985 to 1145 m, with predominantly Litossol and Cambisol soils [25]. According to the Köppen classification, the climate is Cfb type, temperate, with mild summer, annual average temperatures of 17 °C, severe and frequent frosts, and an average rainfall of 1200 mm per year.

The collection and transportation of the plant material were carried out under a license issued by the Paraná Environmental Institute under registration number 284/11. In the collection area, the coordinates were recorded, and a voucher specimen prepared for botanical identification and photographic registration. The voucher specimen was transported to the “Faculdades Integradas Espíritas” Herbarium and registered under protocol number HFIE 8.288.

### 3.2. Essential Oil Extraction and Analysis

The essential oil of the leaves was extracted by hydrodistillation in a Clevenger type apparatus. Briefly, the dried leaves (50 g) were crushed and placed in a 5.0 L glass flask containing 1 L of distilled water, with three repetitions. This material was extracted at boiling temperature for 2.5 h. After extraction, the oil was collected with a precision pipette and stored under refrigeration until testing.

The chemical composition of the oil was analyzed by Gas Chromatography coupled to Mass Spectrometry (GC/MS). The essential oil was diluted in dichloromethane at a rate of 1% and injected in an Agilent 6890 (Palo Alto, CA, USA) chromatograph coupled to an Agilent 5973 N selective mass detector. The injector was kept at 250 °C. The separation of the constituents was obtained by HP-5MS capillary column (5%-phenyl-95%-dimethylpolysiloxane, 30 m × 0.25 mm × 0.25 μm) and using helium as carrier gas (1.0 mL min^−1^). The oven temperature was set at 60 to 240 °C at a rate of 3 °C min^−1^. The mass detector was operated in electronic ionization mode (70 eV) at a rate of 3.15 scan^−1^ and mass range from 40 to 450 u. The transfer line was maintained at 260 °C, the ion source at 230 °C and the analyzer (quadrupole) at 150 °C.

For quantification, the diluted sample was injected into an Agilent 7890 A chromatograph equipped with a flame ionization detector (FID), operating at 280 °C and employing the same column and analytical conditions described above, except for the carrier gas used, which was hydrogen, at a flow rate of 1.5 mL min^−1^. The percentage composition was obtained by the electronic integration of the FID signal by dividing the area of each component by the total area (area%). Identification of components was performed by comparing their mass spectra with the standards reported in the literature [26].

### 3.3. Bacterial Strains

Multiresistant strains of *S. aureus* 10, *P. aeruginosa* 24 e *E. coli* 06, were used throughout this study (Table 2).

### 3.4. Preparation of the Essential Oil Solution

An aliquot of 10 mg of *Croton ceanothifolius* essential oil was dissolved in 1 mL DMSO. Soon after the first dilution, this solution was transferred to a falcon tube and an additional 8.765 mL of sterile distilled water added, making a total of 9.765 mL of solution to a concentration of 1024 µg/mL. This solution was used for minimum inhibitory concentration (MIC) tests and evaluation of the antibiotic-modulating activity in association with Norfloxacin (quinolone), Penicillin (penicillin) and Gentamycin (aminoglycoside) at an initial concentration of 1024 μg/mL.

### 3.5. Determination of the Minimum Inhibitory Concentration (MIC)

Bacterial strains were seeded in Petri dishes containing Heart Infusion Agar (HIA) and kept in the oven at 37 °C for 24 h for growth. After this time, an aliquot of the culture was diluted in test tubes containing sterile saline in triplicate to obtain inocula. The turbidity of the solution was compared to a 0.5 McFarland scale control. A volume of 100 μL of each inoculum (corresponding to 10% of the total solution) was added to a tube containing 900 μL of 10% Brain Heart Infusion (BHI) [27] and then, 100 μL of these solutions were transferred to 96-well plates. The volume of each well was supplemented with 100 μL of the essential oil added after serial dilution at concentrations ranging from 512 μg/mL to 8 μg/mL. All treatments were performed in triplicate.

The plates were incubated at 35 ± 2 °C for 24 h and bacterial growth revealed by the addition of resazurin at 20 μg/mL for 1 h at room temperature. The MIC was visually determined as the lowest concentration capable of inhibiting microbial growth, as attested by the change in color to red [28].

### 3.6. Evaluation of the Antibiotic-Modulating Activity

Volumes of 100 µL of each antibiotic at a concentration of 1024 µg/mL were serially diluted in wells containing 10% BHI and suspensions of the multidrug resistant inocula and essential oil at a subinhibitory concentration (MIC/8) [29]. Controls were prepared by using 1350 µL of 10% BHI medium and 150 µL of the inoculum. Each well of a microdilution plate was filled with 100 µL of the oil or control solution. Following this procedure, serial dilutions with 100 µL the antibiotics at concentrations ranging between 512 and 0.5 μg/mL were carried out. The plates were incubated in an oven at 35 °C for 24 h, and then the MIC of these antibiotics in the presence of the essential oil was determined by the addition of resazurin and interpreted as described above.

### 3.7. Statistical Analysis

Results were expressed as mean ± standard deviation and analyzed by analysis of variance (ANOVA) followed by Bonferroni’s post-test using GraphPad Prism software (GraphPad Software Inc., La Jolla, CA, USA). Differences were considered significant when *p* < 0.05.

## 4. Conclusions

*C. ceanothifolius* essential oil does not have clinically relevant antibacterial activity but has a significant modifying effect on the antibacterial action of some drugs, potentiating their effects against multiresistant bacteria. This is the first study to report the pharmacological properties of this species and, therefore, further studies are fundamental to determine the constituents as well as the mechanisms involved in modulating antibiotic resistance exerted by *C. ceanothifolius* essential oil.

## Figures and Tables

**Table 1:** Chemical constituents identified in the *Croton ceanothifolius* essential oil.

RIa	RIb	Constituent	%
932	937	alpha-pinene	2.8
974	979	beta-pinene	0.8
988	992	Myrcene	0.8
1030	1034	1,8-cineole	0.9
1050	1050	(*E*)-beta-ocimene	0.8
1095	1100	Linalool	1.7
1174	1178	terpinen-4-ol	0.9
1374	1375	alpha-copaene	1.4
1389	1390	beta-elemene	4.2
1417	1417	(*E*)-caryophyllene	11.7
1439	1432	alpha-guaiene	0.9
1452	1451	alpha-humulene	2.4
1458	1458	*allo*-aromadendrene	1.6
1485	1479	germacrene D	14.7
1494	1493	Bicyclogermacrene	26.3
1508	1501	germacrene A	4.7
1522	1521	delta-cadinene	1.3
1577	1574	Spatulenol	2.7
1582	1580	caryophyllene oxide	2.7
1592	1587	Viridiflorol	1.3
1600	1598	Rosifoliol	1.3
1618	1611	1,10-di-epi-cubebol	7.4
1638	1638	epi-alpha-cadinol	2.9
1652	1652	alpha-cadinol	2.9
1770	1761	Squamulosone	0.9
		TOTAL (%)	100

RIa: Literature retention index, RIb: Calculated retention index.

**Table 2:** Resistant profile of the Strains.

Bacteria	Origin	Resistance Profile
*Staphylococcus aureus* 10	Rectal swab	Amc, Amox, Amp, Asb, Azi, Ca, Cef, Cf, Cip, Cla, Clin, Eri, Lev, Mox, Oxa, Pen
*Pseudomonas aeruginosa* 24	Nasal discharge	Ami, Cip, Cpm, Ctz, Imi, Lev, Mer, Ptz
*Escherichia coli* 06	Urine Culture	Asb, Ca, Cef, Cfo, Cmp, Cro

Subtitle: Amc—Amoxicillin + Clavulanic acid, Ami—Amikacin, Amox—Amoxicillin, Amp—Ampicillin, Asb—Ampicillin + Sulbactam, Azi—Azithromycin, Ca—Cefadroxil; Cef—Cephalexin, Cfo—Cefoxitin, Cip—Ciprofloxacin, Cla—Clarithromycin, Clin—Clindamycin, Cmp—Cefepime, Cro—Ceftriaxone, Ctz—Ceftazidime, Eri—Erythromycin, Imi—Imipenem, Lev—Levofloxacin, Mer—Meropenem, Mox—Moxifloxacin, Oxa—Oxacillin, Pen—Penicillin, e Ptz—Piperacillin.
